# Application of multi-task transfer learning: The combination of EA and optimized subband regularized CSP to classification of 8-channel EEG signals with small dataset

**DOI:** 10.3389/fnhum.2023.1143027

**Published:** 2023-03-28

**Authors:** Taixue Long, Min Wan, Wenjuan Jian, Honghui Dai, Wenbing Nie, Jianzhong Xu

**Affiliations:** ^1^The Second Affiliated Hospital, Nanchang University, Nanchang, Jiangxi, China; ^2^Information Engineering School, Nanchang University, Nanchang, Jiangxi, China; ^3^The Army Infantry College of PLA, Nanchang, Jiangxi, China

**Keywords:** EEG, motor imagery, eight channel, VFBRCSP, Common Spatial Pattern

## Abstract

**Introduction:**

The volume conduction effect and high dimensional characteristics triggered by the excessive number of channels of EEG cap-acquired signals in BCI systems can increase the difficulty of classifying EEG signals and the lead time of signal acquisition. We aim to combine transfer learning to decode EEG signals in the few-channel case, improve the classification performance of the motor imagery BCI system across subject cases, reduce the cost of signal acquisition performed by the BCI system, and improve the usefulness of the system.

**Methods:**

Dataset2a from BCI CompetitionIV(2008) was used as Dataset1, and our team's self-collected dataset was used as Dataset2. Dataset1 acquired EEG signals from 9 subjects using a 22-channel device with a sampling frequency of 250 Hz. Dataset2 acquired EEG signals from 10 healthy subjects (8 males and 2 females; age distribution between 21-30 years old; mean age 25 years old) using an 8-channel system with a sampling frequency of 1000 Hz. We introduced EA in the data preprocessing process to reduce the signal differences between subjects and proposed VFB-RCSP in combination with RCSP and FBCSP to optimize the effect of feature extraction.

**Results:**

Experiments were conducted on Dataset1 with EEG data containing only 8 channels and achieved an accuracy of 78.01 and a kappa coefficient of 0.54. The accuracy exceeded most of the other methods proposed in recent years, even though the number of channels used was significantly reduced. On Dataset 2, an accuracy of 59.77 and a Kappa coefficient of 0.34 were achieved, which is a significant improvement compared to other poorly improved classical protocols.

**Discussion:**

Our work effectively improves the classification of few-channel EEG data. It overcomes the dependence of existing algorithms on the number of channels, the number of samples, and the frequency band, which is significant for reducing the complexity of BCI models and improving the user-friendliness of BCI systems.

## 1. Introduction

Brain-computer interface (BCI) technology provides a way of communication that is not dependent on peripheral nerves and muscles (Wolpaw et al., 2000). A comprehensive BCI system involves preprocessing, feature extraction, signal classification, and control. It is a technique that directly translates neurological activity into external output (Ramadan and Vasilakos, 2017). The most commonly employed electroencephalogram (EEG) signals are event-related P300 signals (Allison et al., 2020), steady-state visual evoked potentials (Liavas et al., 1998), and motor imagery (MI) signals (Pfurtscheller et al., 1997). The most significant advantage of motor imagery is that its control signal is derived from the brain's intention to act and therefore does not require external stimulation (Abdulkader et al., 2015). This type of BCI is often used for motion control of external devices and is one of the most popular BCI control systems today. However, the signal-to-noise ratio of motor imagery spontaneous EEG signals is low, and there are significant individual differences in characteristics between subjects. Traditional machine learning algorithms usually need to be calibrated for new subjects to overcome individual differences between subjects (Böttger et al., 2002; Saha et al., 2017), a process that reduces the effectiveness of BCI systems. To address this drawback, researchers have found that using transfer learning algorithms to reduce calibration for new users, devices, and tasks is effective.

In recent years, transfer learning used data or information from the source domain to help the target domain learn by using the source domain (existing subjects) data to calibrate the target domain (new subjects) data (Pan and Yang, 2009). Eventually, the target domain can be judged with few or no samples with annotations, which can solve the problem of mismatch between the base distribution of training data and test data under certain conditions.

Zhang et al. (2021) proposed an adaptive cross-subject transfer learning algorithm based on deep convolutional neural networks to classify new subject data with 84.19% accuracy and high algorithm complexity by analyzing and model training on 62-channel data. Cho et al. (2015) achieved transfer learning of the same subject across experiments by combining Common Spatial Pattern (CSP) and its improved algorithm with Fisher Linear discriminant analysis (LDA). The highest transfer learning result of 79.5% was achieved using 64-channel data. A team from Huazhong University of Science and Technology (He and Wu, 2019) proposed a data alignment preprocessing algorithm EA (Euclidean Alignment, EA) based on Euclidean distance, and the study of two sets of competition data showed that the classification accuracy of most traditional machine learning algorithms was significantly improved after data alignment preprocessing. The two sets of data were 59- and 22-channel, with slight differences in the results of the different algorithms and a maximum accuracy of 79.79% (59-channel). An author in this team proposed a complex transfer learning framework that applies transfer learning in all three aspects of signal preprocessing, feature extraction, and classification, and the analysis of two sets of BCI competition data (59- and 22-channel, respectively) verified that the classification results of this complex framework are higher than those of traditional machine learning. The results of the alignment algorithm with EA data are more significant than transfer learning results without this preprocessing algorithm (Wu et al., 2020a). Jayaram et al. (2016) proposed a Multi-task Transfer Learning (MTL) framework for extracting features shared across experiments and subjects, using Band Power (BP) of 128-channel signals as feature input, with an average classification accuracy of around 76%.

Most of the existing transfer learning algorithms are based on competition data and use data with a large number of channels for analysis (Wu et al., 2020b). Future smart wearable devices based on motor imagery BCI will focus more on portability. Therefore, improving the performance of BCI systems with fewer channels is one of the future research directions to reduce the experimental preparation time and promote the portability of BCIs. In this study, we further investigate reducing calibration and improving the user-friendliness of BCI based on the previous application of CSP to few-channel motor imagery BCI (Dai et al., 2020), and propose an improved algorithm based on transfer learning, VFBRCSP, combining FBCSP and RCSP, and introducing EA processing. We compare the VFBRCSP on the BCI Competition IV Dataset 2a with other new methods proposed in recent years. The results show that with only 8-channel EEG signals, the method proposed in this study still outperforms GRU-RNN (Luo et al., 2018), IST-TSVM (Xu et al., 2019), CA+PSR+CSP (Dong et al., 2020), CSP-WPD+LOG (Zhang et al., 2020), and METL (Cai et al., 2022), achieving a classification accuracy second only to MTFL (Wang et al., 2020). The number of the data's channels used in these BCI systems is 22, which is much more than that of the data we used. From the perspective of reducing the BCI model's complexity and the BCI system's practicality, the method proposed in this study is more advantageous. It effectively improves the classification accuracy in the case of few-channel data, which provides a theoretical and algorithmic basis for further exploring the possibility of commercial implementation of a few-channel motor imagery BCI system.

The structure of this document is as follows: The second section details the experimental dataset used in this study, as well as the data preprocessing procedures, data alignment methods, channel selection scheme, feature extraction methods, and feature classification techniques. In Section 3, the experimental results are given. Section 4 further discusses and analyzes the experimental results. Section 5 presents the conclusions.

## 2. Materials and methods

### 2.1. Data description

The two EEG datasets used in this study are listed below.

#### 2.1.1. Dataset 1

**Figure 1:**
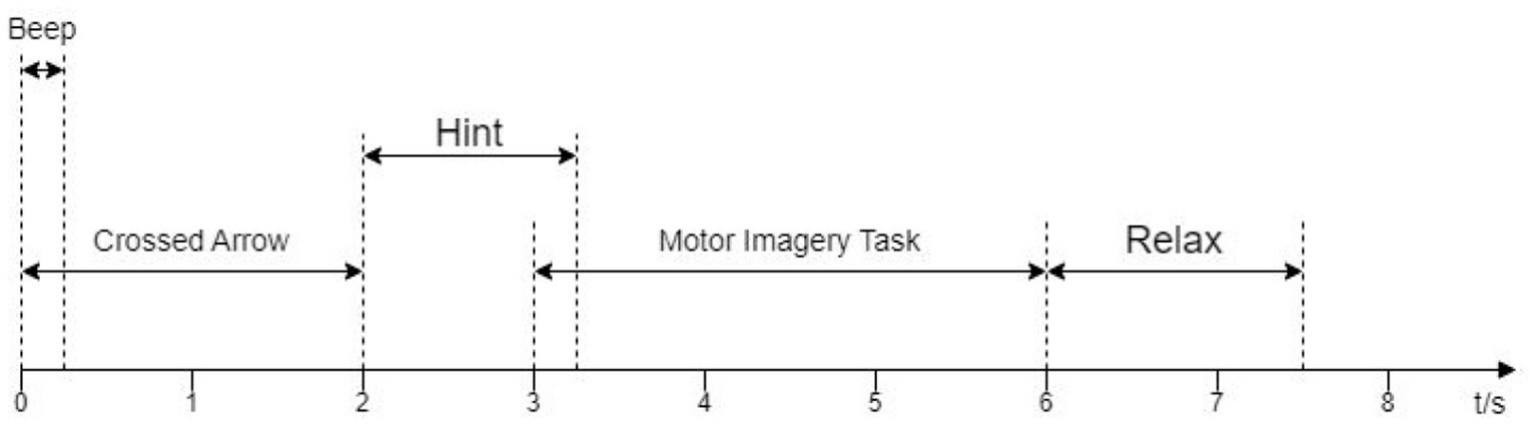
The experimental protocol for the acquisition of Dataset 1.

**Figure 2:**
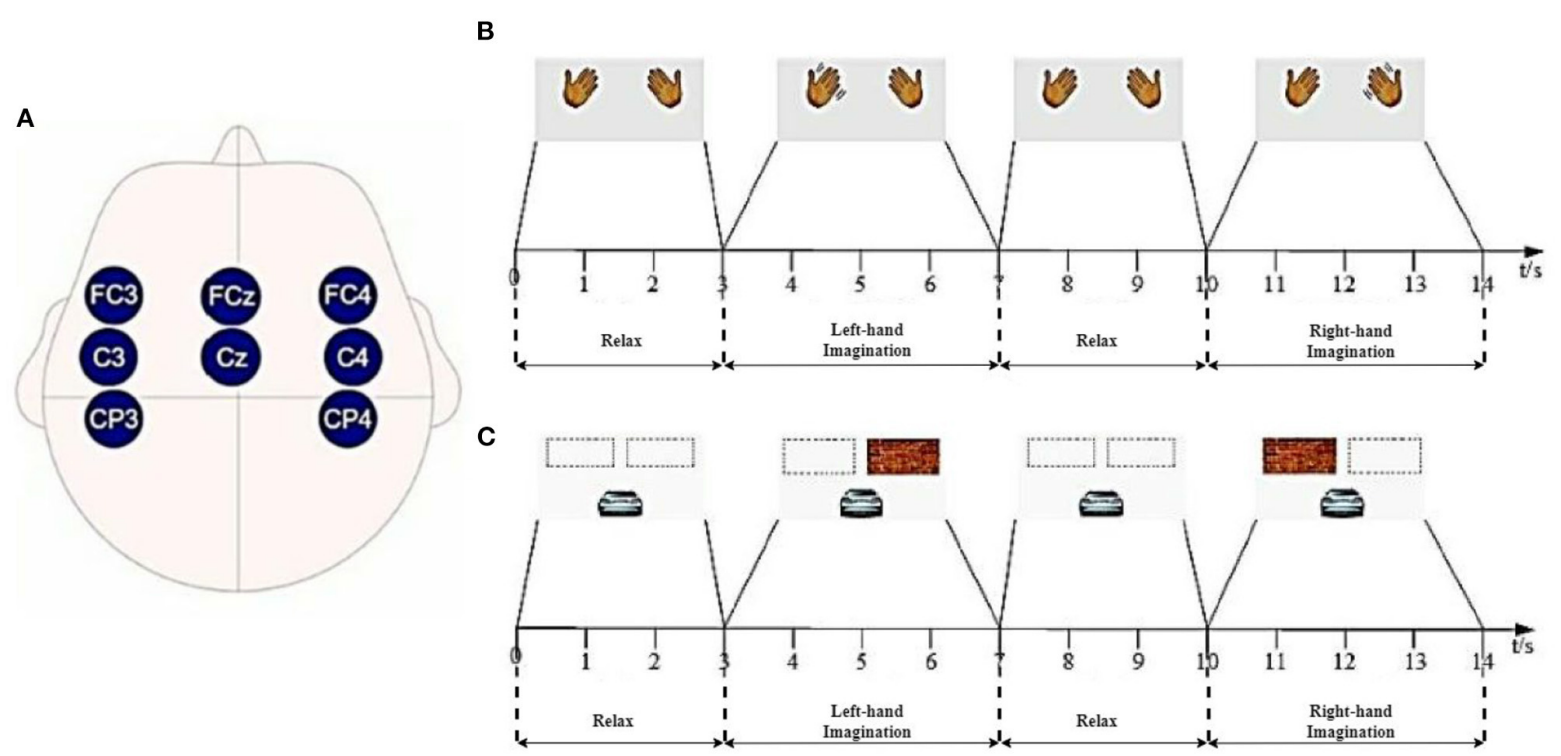
Lead channel distribution and experimental paradigm of Dataset 1: **(A)** Lead distribution; **(B)** Offline experiments; **(C)** Online experiments.

Dataset 2a of BCI CompetitionIV(2008) (Tangermann et al., 2012). The dataset was collected from the EEG signal of nine subjects. All 9 subjects were trained before data collection. Each subject's data were recorded with 22 channels and a sampling frequency of 250 Hz. A total of 288 experiments (containing four categories: right and left hand, foot, and tongue) were performed for each subject. We conducted 144 experiments using the left-hand (Type 1) and right-hand (Type 2) motor imagery data. At the beginning of the experiment, a fixed cross-shaped cursor appeared on the screen with a cue tone, and 2 s later, a pointing arrow appeared on the screen and continued to be displayed for 1.25 s. Subjects performed the corresponding motor imagery task according to the arrow cue, and the motor imagery time was 6 s. At the end of the imagery, subjects rested for around 1.5 s. The specific experimental paradigm is shown in Figure 1. In this study, eight of the 22 channels available for the data in Dataset 1, FC3, FCz, FC4, C3, Cz, C4, CP3, and CP4 (channel distribution locations are shown in Figure 2A), were selected for comparative analysis. Details of the dataset are given in the literature (Tangermann et al., 2012).

#### 2.1.2. Dataset 2

Ten healthy volunteers provided lab-collected data (8 male subjects and 2 female subjects, right-handed, with an age distribution between 21 and 30 years, mean age 25 years). The EEG signal was acquired by wet electrode method, and the acquisition device was NeuSen W wireless digital EEG acquisition system produced by Neuracle Technology (Changzhou) Co., Ltd. with 8 electrode channels (FC3, FCz, FC4, C3, Cz, C4, CP3, and CP4), with CPz as the reference electrode and AFz as the ground electrode, and the sampling frequency was 1,000 Hz, the channel distribution of the acquisition system is shown in Figure 2A. For each subject, the experiment involved two sections, offline and online, with offline training data including no feedback and online training data containing feedback. Figure 2B shows the offline experiment paradigm. Figure 2C shows the online experiment paradigm.

### 2.2. Proposed method

**Figure 3:**
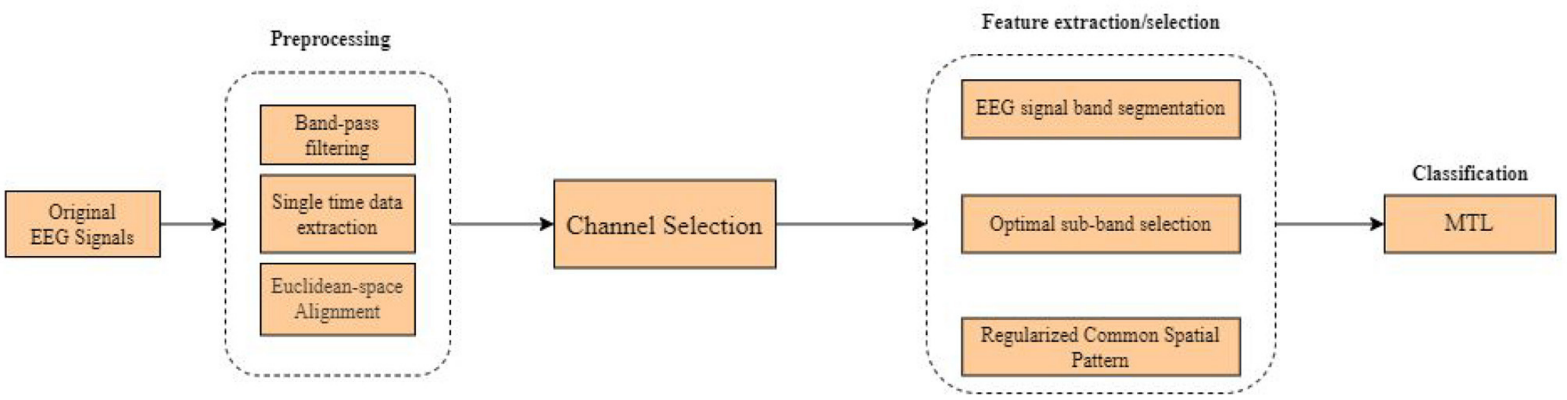
The processing flow of the proposed method.

Figure 3 shows the flow chart of the overall processing of the proposed method, which mainly consists of preprocessing, data alignment, channel selection, feature extraction, and classification. Each part will be discussed in detail in the following sections.

#### 2.2.1. Data preprocessing

##### 2.2.1.1. Band-pass filtering

The ERS/ERD phenomenon that occurs during motor imagery causes power variations in specific frequency bands of the EEG signal, which usually occur in the two frequency bands of mu (8–12 Hz) and beta (18–25 Hz) rhythms (Lazarou et al., 2018). Therefore, in the proposed work, a 6th-order Butterworth filter is used to band-pass the EEG signal from 8 to 30 Hz to filter out extraneous components of the EEG signal.

##### 2.2.1.2. Extraction of single time data

This experiment's signal was retrieved 0.5 s after the subject accepted the experimental hint. Each motor imagery experiment began at 0 s. Hence Dataset 1 was cropped for 0.5–3.5 s and Dataset 2 for 0.5–4 s.

##### 2.2.1.3. Data alignment

The Euclidean-space Alignment (EA) proposed by He et al. was used for data alignment of the dataset. This algorithm directly aligns the original data of EEG samples from different subjects while maintaining the original data structure of the EEG samples (He and Wu, 2019), thus improving the similarity of EEG signal distribution across subjects.

#### 2.2.2. Channel selection

We manually selected the channel locations according to the prior knowledge of the brain-computer interface domain. Dataset 1 was acquired from a 64-channel system expanded by 10-20 international standard channel systems. The data acquired by the eight channels shown in Figure 2A were selected based on the a priori knowledge of physiology in the field of motor imagery and previous studies of our team (Jian et al., 2017a,b), and Dataset 2 was acquired directly using the same eight channels for data acquisition.

#### 2.2.3. EEG signal band segmentation

**Figure 4:**
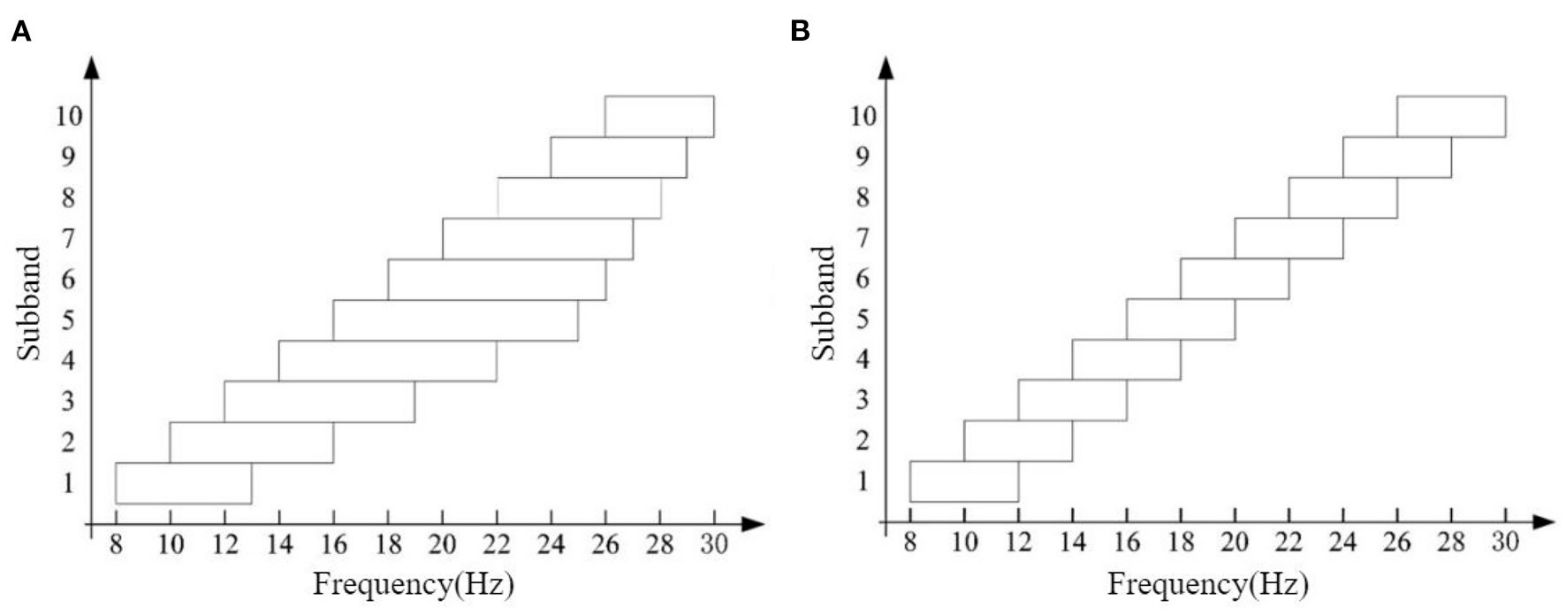
Frequency band selection scheme: **(A)** VFB and **(B)** CFB.

Considering the differences in the optimal frequency bands of different subjects (Chen et al., 2015), in order to utilize the effective frequency bands as much as possible, this study first designed a band-splitting scheme with fixed sub-band bandwidth: Constant Filter Bank (CFB). This scheme divides the EEG signal into 10 subbands in the range of 8–30 Hz for filtering, and the bandwidth of each sub-band is 4 Hz, and the adjacent subbands overlap by 2 Hz, as shown in Figure 4B. This scheme is abbreviated as CFB in the later paper, and the feature extraction method using this scheme for band segmentation is abbreviated as CFB-RCSP. Based on CFB, we try to vary the sub-band bandwidth and further propose a Variable Filter Bank (VFB) as the final band segmentation scheme: the first sub-band starting at 8 Hz, and then each sub-band starting at 2 Hz, and the bandwidths of each sub-band are 5, 6, 7, 8, 9, 8, 7, 6, 5, and 4 Hz (first from narrow to wide, and then The bandwidth of each sub-band is 5, 6, 7, 8, 9, 8, 7, 6, 5, and 4 Hz (first from narrow to wide and then from wide to narrow), as shown in Figure 4A.

#### 2.2.4. Optimal subband selection

After the frequency domain filtering of the EEG signals using the band splitting scheme proposed in Section 2.2.3, these EEG signals are split into several sub-band signals with different bandwidths. The sub-band features are extracted from these sub-band signals by space domain filtering, and the space domain filtered features have optimal space domain information, but there is redundancy in the frequency domain, so the Fisher score method combined with grid search is introduced for optimal frequency band selection (Nie et al., 2008). The basic idea of optimal frequency band selection is:

Suppose the eigenvector of each sub-band signal *x*_*i*_ is (*x, l*) ∈(*R*^*d*^×*L*), where *R*^*d*^ is the feature space, *L* represents the feature class label, and there are *k* categorical features (*k*∈{1, −1} in this study, representing the left and right hands respectively), the intra-class scatter matrix *S*_*W*_ and inter-class scatter matrix *S*_*B*_ of the eigenvector are:


(1) $$\begin{array}{l} S_{B}=\overset{k}{\underset{i=1}{\sum }}n_{i}\left(m_{i}-m\right){\left(m_{i}-m\right)}^{T} \end{array}$$

(2) $$\begin{array}{l} S_{W}=\overset{k}{\underset{i=1}{\sum }}\overset{n_{i}}{\underset{j=1}{\sum }}\left(x_{ij}-m_{i}\right){\left(x_{ij}-m_{i}\right)}^{T} \end{array}$$

In Equations (1), (2), *n*_*i*_ denotes the sample size of the *i*-th class of features, *m*_*i*_ represents the average vector of the *i*-th class of feature vectors, and *m* is the average vector of all eigenvectors.

According to the classification criteria, the Fisher score for each sub-band can be defined as:


(3) $$\begin{array}{l} f_{s}=\frac{Tr\left(S_{B}\right)}{Tr\left(S_{W}\right)} \end{array}$$

The *f*_*s*_ of each sub-band can be calculated by Equation (3), but considering that the band power of the EEG signal decreases when its frequency increases, the squared weighted value ξ of *f*_*s*_ is used as the optimal sub-band selection criterion in combination with the grid search. The expression of ξ is given by:


(4) $$\begin{array}{l} \xi_{n}=w\left(n\right){\left(f_{s}^{n}\right)}^{2},n\in \left[1,N\right] \end{array}$$

In Equation (4), $f_{s}^{n}$ is the Fisher score of the *n*-th sub-band. *w*(*n*) is the weight value, which can be calculated by Equation (5):


(5) $$\begin{array}{l} w\left(n\right)=n^{-a}+b \end{array}$$

For each sub-band *n*, the weight value *w*(*n*) is transformed with parameters *a* and *b*, and ξ_*n*_ varies with *w*(*n*). The corresponding sub-band is the optimal sub-band when the value of ξ_*n*_ is maximum.

#### 2.2.5. Regularized common spatial pattern

The traditional CSP algorithm finds a set of spatial filters to maximize the variance of one class while minimizing the variance of another class, thus obtaining a feature vector with a high degree of discrimination.

In the case of the dichotomous EEG imagery task, suppose the target subject has *N* samples ${\left\{X_{t}^{n}\right\}}_{n=1}^{N}$. For the category *k*∈{−1, 1}, the CSP tries to find the spatial filter matrix $W_{k}\in {\mathbb{R}}^{c\times f}$ maximizing the variance ratio between the two categories, where *c* is the number of channels of the EEG signal and *f* is the number of spatial filters:


(6) $$\begin{array}{l} W_{k}=\arg\max\frac{Tr\left(W^{T}{\bar{C}}_{t}^{k}W\right)}{Tr\left(W^{T}{\bar{C}}_{t}^{-k}W\right)} \end{array}$$

In Equation (6), ${\bar{C}}_{t}^{-k}\in {\mathbb{R}}^{c\times c}$ is the average spatial covariance matrix of the EEG signals in the target subjects with category *K*.

In order to reduce the differences in the distribution of features between subjects, RCSP helps train the feature values with the help of partial source domain subject data based on the CSP method. With (7), RCSP can obtain the regularized mean covariance matrix of the EEG signal:


(7) $$\begin{array}{l} {Ĉ}^{k}\left(\beta ,\gamma \right)=\left(1-\gamma \right){Ĉ}^{k}\left(\beta \right)+\frac{\gamma }{c}\left({Ĉ}^{k}\left(\beta \right)\right)I \end{array}$$

In Equation (7), β and γ are two regularization parameters located in the interval [0, 1]. β is used to reduce the variance of the sample covariance matrix estimates, and γ is used to control the degree of contraction of the unit matrix. *I*∈*R*^*c*×*c*^ is a unit matrix, and Ĉ^*k*^(β) can be derived from Equation (8).


(8) $$\begin{array}{l} {Ĉ}^{k}\left(\beta \right)=\frac{\beta N_{l}{\bar{C}}_{t}^{k}+\left(1-\beta \right)N_{s}{\bar{C}}_{s}^{k}}{\beta N_{l}+\left(1-\beta \right)N_{s}} \end{array}$$

In Equation (8), ${\bar{C}}_{s}^{k}\in R^{c\times c}$> is the mean spatial covariance matrix of the EEG samples originating from the subjects. The RCSP method is obtained by replacing ${\bar{C}}_{t}^{k}$ with Ĉ^*k*^(β, γ) in Equation (6).

To conduct RCSP in our MI-BCI system, we treat the labels and divide the dataset, the detailed description is as follows:

1. Label Definition:

- Dataset 1 (BCI Competition IV dataset 2a): The left and right hand MI data in the training session of each subject in Dataset 1 were used. The left hand MI data is defined as the first category, and the right hand MI data is defined as the second category;
- Dataset 2 (self-collected dataset): All data obtained in offline and online experiments are used, and the left and right hands are also used as labels (first and second).

2. Dataset Division:

- Dataset 1 and Dataset 2: To conduct RCSP, based on the 8-lead EEG signal, one subject was selected as the target subject (test set) in turn by using the one-left method, and the data of the other subjects were merged into the source subjects (training set). RCSP is a feature extraction algorithm of EEG signals based on Riemannian space. In order to reduce the difference of feature distribution between different subjects, RCSP, based on the CSP method, extracts feature values with the help of source domain subject data. It extracts the best feature dimensions from the source domain dataset during the modeling process, and uses these feature dimensions to represent the target subject data: First, it calculates the eigenvectors of the subjects in the source domain through different subspace methods. Then, the matching ratio between the eigenvector of subjects in the target domain and the eigenvector of subjects in the source domain is calculated by the original EEG signal and the EEG signal obtained from the subspace mapping. Finally, it calculates the weight of each feature dimension according to the matching ratio, so as to extract representative features from the target subject data.

#### 2.2.6. MTL classification

The proposal of MTL classification algorithm is derived from the idea of transfer learning. Since the EEG signal is not static, in the strictest sense, each experiment can be condsidered as a slightly new task relative to each other, which means that either the classification task of EEG data from different subjects or the classification task of EEG signals from the same subjects under different conditions (distinguishing between left and right hand) can be considered as separate tasks. In the experiments, the raw data are classified by the MTL classification algorithm proposed by Jayaram et al. (2016) after the data preprocessing process described in Section 2.2.1 and the band segmentation and feature extraction. The MTL algorithm takes one subject's data as the target domain and the other subjects' data as the source domain in turn during each experiment. The classification performance on the target domain is optimized by training in the source domain, hence the BCI system proposed in this study can be regarded as cross-subject. MTL algorithm allows our BCI system to use the information from all tasks to improve the cclassification model for each task and obtain a shared structure as a priori information, which will ensure that the solutions of all tasks are sufficiently close to each other in a certain space. Finally, we get the optimal classification model relative to the whole dataset for classification. The actual optimization problem can be defined as Jayaram et al. (2016):


(9) $$\begin{array}{l} \;\min LP(W,\mu ,\Sigma ;D,\lambda )=\\ \min\frac{1}{\lambda }\sum_{s}{\left\|F_{s}w_{s}-y_{s}\right\|}^{2}+\sum_{s}\Omega (w_{s};\mu ,\Sigma ) \end{array}$$

In Equation (9), $W={\left[w_{1},w_{2},\ldots ,w_{s}\right]}^{T}$ denotes the feature weight parameter matrix, μ denotes the mean vector of subject features, Σ denotes the feature covariance matrix of subjects, $D={\left\{D_{s}\right\}}_{s=1}^{S}$ denotes the total of all subject data, *s* is the subject number, λ denotes the standard deviation of model noise, *F*_*s*_ is the feature matrix of subject s, *w*_*s*_ is the linear classifier weight, *F*_*s*_*w*_*s*_ denotes the predicted label, and *y*_*s*_ is the actual label. Ω(·) denotes the penalty term used to reduce the complexity of the model, which is calculated as:


(10) $$\begin{array}{l} \;\Omega (w_{s};\mu ,\Sigma )=\\ \frac{1}{2}[{\left(w_{s}-\mu \right)}^{T}\Sigma^{-1}(w_{s}-\mu )+\frac{1}{2}\log\;\det(\Sigma )] \end{array}$$

Equation (10) controls the difference in the average vector of features μ for each subject. The feature weight parameter *w*_*s*_ is iterated as shown in Equation (11).


(11) $$\begin{array}{l} \begin{array}{c} w_{s}={\left(\frac{1}{\lambda }\Sigma F_{s}^{T}F_{s}+I\right)}^{-1}\left(\frac{1}{\lambda }\Sigma F_{s}^{T}y_{s}+\mu \right) \end{array} \end{array}$$

where *I* is the unit matrix and *w*_*s*_ is jointly determined by the eigenmean vector μ, the eigencovariance matrix Σ and the product of subject eigenmatrices $F_{s}^{T}F_{s}$.

## 3. Results

### 3.1. Evaluation of feature extraction method optimization

The scheme in which the band energy of the EEG signal is directly extracted as features is marked as BP; the schemes in which band-pass filtering is first applied to the EEG signal and then the RCSP, FBCSP, CFB-RCSP, and VFB-RCSP are used for feature extraction are denoted as RCSP, FBCSP, CFB-RCSP, and VFB-RCSP, respectively.

When comparing the performance of each scheme, the classification accuracy served as the primary evaluative metric. In addition, we used the Kappa coefficient (Cohen, 1960, 1968) as another evaluation index to prevent the unbalanced sample condition caused by the limited sample size from affecting the experimental results.

The values presented in Table 1 are the classification accuracies and Kappa coefficients obtained for the different schemes on the data of all subjects in Dataset 1, where the values of Kappa coefficients are shown in parentheses, and the optimal classification accuracies are bolded in the table. According to the data presented in Table 1, VFB-RCSP achieved the highest average classification accuracy and kappa coefficient among the various schemes involved in the comparison, and the highest classification accuracy and optimal kappa coefficient were achieved on the data of more than half of the subjects.

**Table 1:** Classification accuracy of the proposed method and other new methods on Dataset 1.

**Subject**	**BP**	**RCSP**	**FBCSP**	**CFB-RCSP**	**VFB-RCSP**
1	80.56 (0.54)	82.86 (0.58)	70.83 (0.40)	**86.81 (0.65)**	86.11 (0.63)
2	52.43 (0.23)	59.64 (0.29)	**76.39 (0.48)**	64.58 (0.34)	70.83 (0.41)
3	67.01 (0.36)	93.57 (0.80)	78.47 (0.51)	**94.44 (0.82)**	**94.44 (0.83)**
4	67.01 (0.37)	**76.07 (0.47)**	71.53 (0.41)	74.31 (0.45)	73.61 (0.44)
5	55.56 (0.26)	56.79 (0.27)	60.42 (0.30)	59.72 (0.29)	**61.11 (0.30)**
6	63.89 (0.33)	68.93 (0.38)	72.22 (0.42)	**75.69 (0.47)**	70.83 (0.41)
7	64.58(0.34)	65.36 (0.35)	**66.67 (0.36)**	64.58 (0.34)	63.89 (0.33)
8	74.65 (0.45)	87.86 (0.67)	73.96 (0.44)	92.36 (0.77)	**93.06 (0.79)**
9	71.88 (0.42)	83.21 (0.58)	72.92 (0.43)	86.81 (0.65)	**88.19 (0.69)**
Mean	66.40 (0.37)	74.92 (0.49)	71.49 (0.42)	77.70 (0.53)	**78.01 (0.54)**

The value of the highest classification accuracy obtained by various methods on the data of each patient is displayed in bold.

Table 2 shows the classification accuracy of the data of all subjects in Dataset 2 under different schemes. Among the various classification methods, the highest average classification accuracy and Kappa coefficient were obtained for the proposed method in this chapter. The highest classification accuracy and Kappa coefficient were obtained for most of the subjects. The highest classification accuracy is bolded in the table.

**Table 2:** Classification accuracy of the proposed method and other new methods on Dataset 2.

**Subject**	**BP**	**RCSP**	**FBCSP**	**CFB-RCSP**	**VFB-RCSP**
1	55.59 (0.29)	55.57 (0.29)	56.08 (0.30)	**62.83 (0.37)**	60.86 (0.35)
2	55.26 (0.29)	54.14 (0.28)	56.76 (0.31)	57.24 (0.31)	**61.84 (0.36)**
3	55.92 (0.30)	53.97 (0.28)	55.41 (0.29)	**60.86 (0.35)**	60.53 (0.34)
4	54.93 (0.29)	54.9 (0.29)	54.73 (0.29)	55.92 (0.30)	**56.58 (0.31)**
5	57.23 (0.31)	54.39 (0.28)	55.41 (0.30)	**57.89 (0.32)**	57.24 (0.31)
6	57.56 (0.32)	55.41 (0.30)	56.76 (0.31)	57.24 (0.31)	**62.5 (0.36)**
7	**63.48 (0.37)**	53.55 (0.28)	59.46 (0.33)	62.5 (0.36)	59.21 (0.33)
8	55.59 (0.30)	55.15 (0.29)	58.11 (0.32)	**61.84 (0.36)**	57.89 (0.32)
9	55.26 (0.30)	55.41 (0.30)	57.43 (0.31)	**59.87 (0.34)**	59.21 (0.31)
10	56.57 (0.30)	55.57 (0.30)	56.08 (0.30)	59.21 (0.33)	**61.84 (0.33)**
Mean	56.74 (0.31)	54.81 (0.29)	56.623 (0.30)	59.54 (0.33)	**59.77 (0.34)**

The value of the highest classification accuracy obtained by various methods on the data of each patient is displayed in bold.

**Figure 5:**
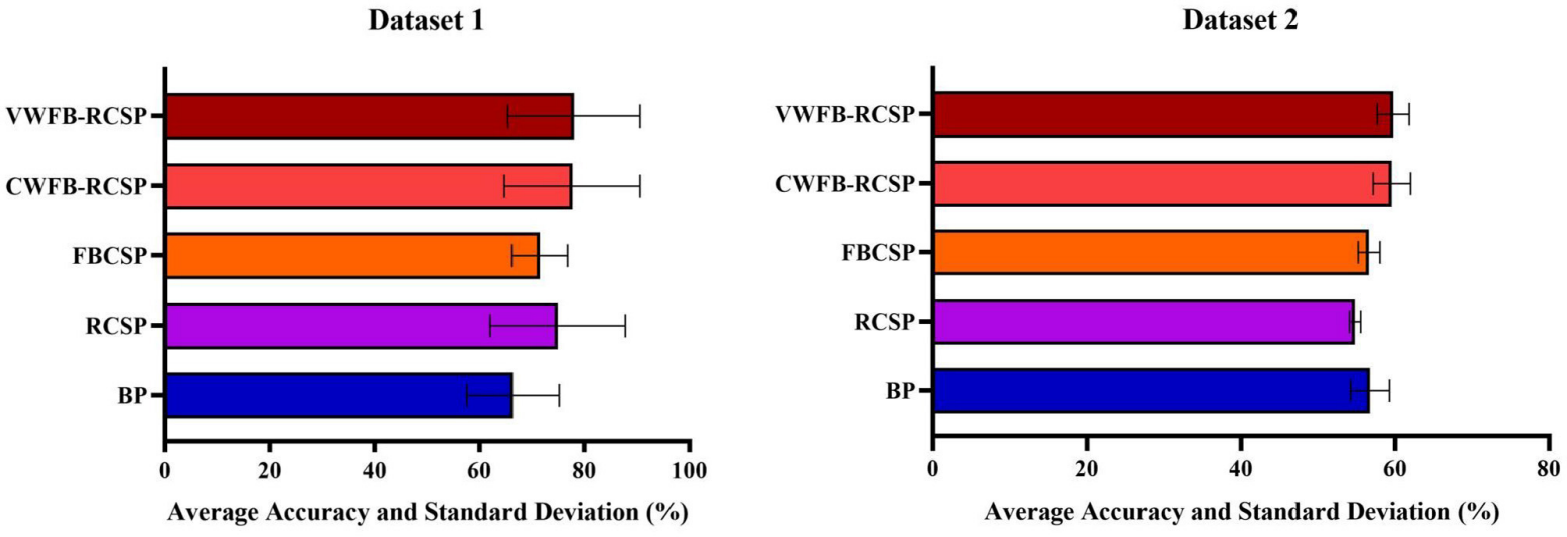
Average classification accuracy and standard deviation of Dataset 2 under different methods.

Figure 5 visualizes the two datasets' average classification accuracy and standard deviation under different methods. In Dataset 1, the average classification accuracy and standard deviation for each method are:*BP*(66.40 ± 8.81), *RCSP*(74.92 ± 12.93), *FBCSP*(71.49 ± 5.33), *CFB*−*RCSP*(77.70 ± 12.96), *VFB*−*RCSP*(78.01 ± 12.61). The average classification accuracy and standard deviation of each method in Dataset 2 are:*BP*(56.73 ± 2.52), *RCSP*(54.81 ± 0.74), *FBCSP*(56.62 ± 1.42), *CFB*−*RCSP*(59.54 ± 2.43), *andVFB*−*RCSP*(59.77 ± 2.06). The *VFB*−*RCSP* proposed in this study achieves the best results in both datasets with respect to other pre-improvement schemes.

The statistical results of the one-way repeated measures ANOVA on the classification results of the two datasets with the “feature extraction metho” as a factor showed that the main effect of the “feature extraction method” was significant in both Dataset 1 [*F*_(3, 51)_ = 44.64, *p* < 0.0001] and Dataset 2 [*F*_(1, 106)_ = 11.53, *p = 0.6832*, *F*_(5, 40)_ = 15.17, *p* < 0.0001]. Table 3 shows the comparison of CFB-RCSP and VFB-RCSP with the other three methods under the two datasets, where the results with significant differences are bolded in the table. The results of Tukey's *post hoc* test showed that in Dataset 1, the classification accuracy of CFB-RCSP and VFB-RCSP is significantly higher than that of BP. In Dataset 2, the classification accuracy of both CFB-RCSP and VFB-RCSP methods was significantly higher than that of the other three methods.

**Table 3:** One-way repeated measures ANOVA statistical test results, *p* < 0.05 means that the results are significant.

	**Dataset 1**			**Dataset 2**		
**Algorithm**	**BP**	**RCSP**	**FBCSP**	**BP**	**RCSP**	**FBCSP**
*CFB-RCSP*	***p*** **=** **0.0016**	*p* = 0.8350	*p* = 0.1638	***p*** **=** **0.0109**	***p*** ** < 0.0001**	***p*** **=** **0.0074**
*VFB-RCSP*	***p*** **=** **0.0012**	*p* = 0.7763	*p* = 0.1311	***p*** **=** **0.0050**	***p*** ** < 0.0001**	***p*** **=** **0.0034**

The value of the highest classification accuracy obtained by various methods on the data of each patient is displayed in bold.

The combined classification and statistical test results reveal that the suggested method outperforms the other unimproved methods on both datasets. VFB-RCSP performs slightly better than CFB-RCSP with a fixed bandwidth at subband segmentation in both datasets, but the difference is not statistically significant.

### 3.2. Evaluation of EEG motor imagery signal classification performance

To more comprehensively evaluate the effectiveness of the proposed method in this study, we compared the classification accuracy of the proposed method VFB-RCSP with some methods proposed in recent years on Dataset 1, and the results are shown in Table 4. However, compared to MTFL, the proposed VFB-RCSP was performed on 8-channel data, and the number of channels used decreased significantly compared to the 22 channels used in MTFL while the classification accuracy was not reduced much.

**Table 4:** Classification accuracy of the proposed method and other new methods on Dataset 1.

**Subjects**	**GRU-RNN** **(2018)**	**IST-TSVM** **(2019)**	**CA+PSR+CSP** **(2020)**	**MTFL** **(2020)**	**CSP-WPD+LOG** ** (2020)**	**METL** **(2022)**	**Ours**
Lead channels	22	22	22	22	22	22	**8**
1	84.82	80.14	80.00	91.67	81.25	88.19	86.11
2	65.32	51.55	65.36	63.19	63.79	56.26	70.83
3	83.54	95.54	87.14	95.14	90.97	97.91	94.44
4	67.67	53.60	67.50	72.22	70.13	74.30	73.61
5	64.00	51.65	55.54	64.58	53.47	59.03	61.11
6	70.87	56.83	50.18	68.06	61.81	70.83	70.83
7	84.96	56.58	91.79	79.17	60.42	71.52	63.89
8	71.95	93.42	84.11	97.92	84.72	91.67	93.06
9	68.90	92.66	87.86	92.37	75.69	81.25	88.19
Mean ± std	73.56 ± 4.38	70.22 ± 19.74	74.39 ± 15.18	**80.48** **±** **13.97**	71.29 ± 12.59	76.77 ± 14.25	**78.01** **±** **12.61**

The value of the highest classification accuracy obtained by various methods on the data of each patient is displayed in bold.

## 4. Discussion

The results presented in Section 3 show the superiority of the proposed scheme, a new method for classifying few-channel motor imagery EEG signals that somewhat increase the classification accuracy of the few-channel motor imagery BCI system.

In Section 1, we have introduced the problem of excessive number of electrode leads in the acquisition system, which is an urgent problem to be solved in recent years for the practicalization of BCI systems. At the same time, the small number of samples is a significant problem for motor imagery signal classification. We combine transfer learning with data preprocessing and feature extraction to optimize these two problems. In the data preprocessing process, EA was introduced to reduce the signal differences among subjects to achieve the desired effect of transfer learning better. Meanwhile, the optimal subbands of each subject group in Dataset 1 and Dataset 2 were filtered based on Fisher score combined with grid search to evaluate the optimization of band selection on the feature extraction process. The results are shown in Tables 5, 6. Obviously, the optimal subbands are different for different subjects, so band selection for each subject will help improve the classification performance of the motor imagery BCI system. According to Table 5, the optimal subbands of these trained subjects in Dataset 1 were mostly located at 16–26 Hz, which basically coincided with the beta rhythm (18–25 Hz). Table 6 shows that the optimal frequency bands of the untrained subjects in Dataset 2 are unevenly distributed between 8 and 26 Hz, which further illustrates the importance of band selection despite the discrepancy with the results in Dataset 1. Moreover, compared to the fixed-bandwidth CFB-RCSP, the VFB-RCSP, which varies the sub-band bandwidth within the optimal sub-band range, has a better chance of obtaining the optimal sub-band range of different subjects, as also shown in Section 3.1. Meanwhile, considering the average classification accuracy achieved on Dataset 1 and Dataset 2 (78.01% in Dataset 1 and 59.77% in Dataset 2), we believe that the distribution of EEG signals in the EEG cortex of subjects without training may be more extensive, resulting in the poor quality of EEG signals acquired by the 8-channel-based motor imagery EEG signal acquisition system to meet the subsequent classification requirements. Therefore, the following research will focus on improving the feature extraction algorithm and enhancing the BCI system's classification performance for data from untrained subjects in the case of fewer channels.

**Table 5:** Optimal subband range of subjects in Dataset 1.

**Subject**	**Optimal subband range (Hz)**	
	**CFB**	**VFB**
A1	20–24	18–26
A2	18–22	18–26
A3	20–24	16–25
A4	20-24	18–26
A5	10–14	10–16
A6	20–24	18–26
A7	18–22	16–25
A8	18–22	18–26
A9	18–22	18–26

**Table 6:** Optimal subband range of subjects in Dataset 2.

**Subject**	**Optimal subband range (Hz)**	
	**CFB**	**VFB**
S1	20–24	18–26
S2	8–12	8–13
S3	20–24	16–25
S4	18–22	10–16
S5	10–14	10–16
S6	20–24	18–26
S7	10–14	10–16
S8	18–22	18–26
S9	18–22	18–26
S10	10–14	10–15

According to Table 3, the performance of CFB-RCSP and VFB-RCSP proposed in this study on Dataset 1 is not significantly improved compared with RCSP and FBCSP, but it is still significantly improved compared with BP. In contrast, the CFB-, VFB-RCSP proposed in this study has a significant improvement compared with RCSP and FBCSP in Dataset 2. It is worth mentioning that the subjects in Dataset 1 have been trained for a period of time and have rich experience in BCI collection experiments, while the information collected in Dataset 2 used in this study comes from subjects without any training. The experiment on Dataset 2 is more in line with the situation of using BCI system in daily life. From the fact that the performance of Dataset 2 is significantly better than that of algorithms such as RCSP, FBCSP and BP, but the performance improvement of Dataset 1 is insufficiently significant compared with algorithms such as RCSP and FBCSP, it can be inferred that the improvement made in this study reduces the requirements of BCI system for users' training time to a certain extent, and is more friendly to the popularization and practicality of BCI system, which is beneficial for BCI system to go out of the laboratory and into people's daily life.

In addition, the algorithm proposed in this paper improves the classification performance of the motor imagery task with few channels by filtering the optimal band information through filter bank splitting subbands and generating a regularized covariance matrix by introducing the source subject EEG signal. However, this also causes an increase in the overall system completion time for classification. In future work, there is a need for in-depth research on how to improve computational efficiency while maintaining the classification performance of this algorithm.

Finally, according to the results in Section 3.2, although the VFB-RCSP scheme proposed in this paper does not achieve the highest classification accuracy compared to a series of methods proposed in recent years, considering that the experiments in this study are based on 8-channel EEG signals, while all other methods in Table 4 are based on 22-channel EEG signals. In other words, the classification accuracy of this method can still compare with and surpass the majority of these 22-channel EEG-based schemes in Table 4 by achieving the second-highest classification accuracy despite the significant reduction in the number of channels. Therefore, the proposed method is more advantageous from the perspective of reducing the BCI model's complexity and the BCI system's practicality.

## 5. Conclusion

Existing algorithms depend on the number of channels, samples, and frequency bands. In this paper, we apply transfer learning to a BCI system for motor imagery signal classification, propose an 8-channel scheme based on brain science, combine optimal subbands and regularized filter bank co-space patterns to propose VFB-RCSP, and perform EA alignment on the data during experiments. Our BCI system produced substantial results in categorizing 8-channel motor imagery EEG signals and enhanced classification performance over the original method and other current algorithms. Our BCI system had an outstanding average classification accuracy of 78.01% on the BCI competition IV 2a 8-channel EEG dataset. The highest average classification accuracy of 59.77 was also achieved on Dataset 2, which was acquired independently by our team. This result is a significant improvement compared to various classical algorithms without improvement, which validates the effective improvement of our proposed improvement for the classification performance of the few-channel motor imagery EEG signal system. We will continue researching and optimizing the classification of few-channel motor imaging signals. The feature extraction technique can be modified to optimize the quality of the EEG signals recorded by the 8-channel-based motor imagery EEG signal acquisition system; computational efficiency can be improved while retaining classification performance. Future study will also include the multi-classification task of motor imagery EEG signals.

## Data availability statement

The raw data supporting the conclusions of this article will be made available by the authors, without undue reservation.

## Ethics statement

The studies involving human participants were reviewed and approved by the Second Affiliated Hospital of Nanchang University Medical Research Ethics Committee. The patients/participants provided their written informed consent to participate in this study.

## Author contributions

TL, WJ, MW, and HD conceived and designed the whole research. TL, HD, WN, and JX took responsibility for the integrity of the data and the accuracy of the data analysis. WJ and HD collected the data. TL wrote the main manuscript text. TL, MW, HD, WN, and JX took responsibility for the statistical data analysis and the critical interpretation of the data. All authors contributed to the final version of the manuscript and have read and approved the final manuscript.
